# Cardiac and hepatic phenotype of diabetes in the presence and the absence of obesity - mechanistic role of ectopic/visceral adiposity

**DOI:** 10.1186/1532-429X-18-S1-P224

**Published:** 2016-01-27

**Authors:** Eylem Levelt, Michael Pavlides, Masliza Mahmod, Catherine Kelly, Joanne Sellwood, Sheena Thomas, Jane M Francis, Jurgen E Schneider, Chris Rodgers, William T Clarke, Nikant Sabharwal, Charalambos Antoniades, Kieran Clarke, Theodoros D Karamitsos, Oliver Rider, Stefan Neubauer

**Affiliations:** 1 grid.4991.50000000419368948OCMR, University of Oxford, Oxford, United Kingdom; 2 Perspectum Diagnostics Ltd, Oxford, United Kingdom; 3 grid.8348.70000000123067492Division of Cardiovascular Medicine, John Radcliffe Hospital, Oxford, United Kingdom; 4 grid.4991.50000000419368948Department of Physiology, Anatomy & Genetics, University of Oxford, Oxford, United Kingdom

## Background

Ectopic/visceral adiposity is frequently observed in type 2 diabetes mellitus (T2DM) and is linked to cardiovascular mortality. Non-alcoholic fatty liver disease (NAFLD) is an example of ectopic fat accumulation in a visceral organ, also strongly linked with cardiovascular mortality. We assessed the diabetes-associated cardiac and hepatic changes, and hypothesised that they will be amplified by the co-existence of obesity, and that the ectopic adiposity may play a pathophysiological role in the cardiac and hepatic phenotype of diabetes.

## Methods

Twenty-seven obese T2DM (O-T2DM) patients, fifteen lean T2DM (L-T2DM) patients, and twelve healthy volunteers were studied. T2DM patients underwent cardiac CT (epicardial fat quantification and exclusion of significant CAD), cardiac MRI (cine and tagging), ^1^H-, ^31^P-MRS for myocardial triglyceride (MTG) and PCr/ATP respectively, and a multi-parametric liver MRI scan, including ^1^H-MRS for hepatic triglyceride (HTG), T1 and T2* mapping yielding an ‘iron-corrected T1' (cT1), a parameter which allows non-invasive quantification of fibroinflammatory liver disease. Healthy volunteers underwent identical MRI protocols.

## Results

**Figure 1:**
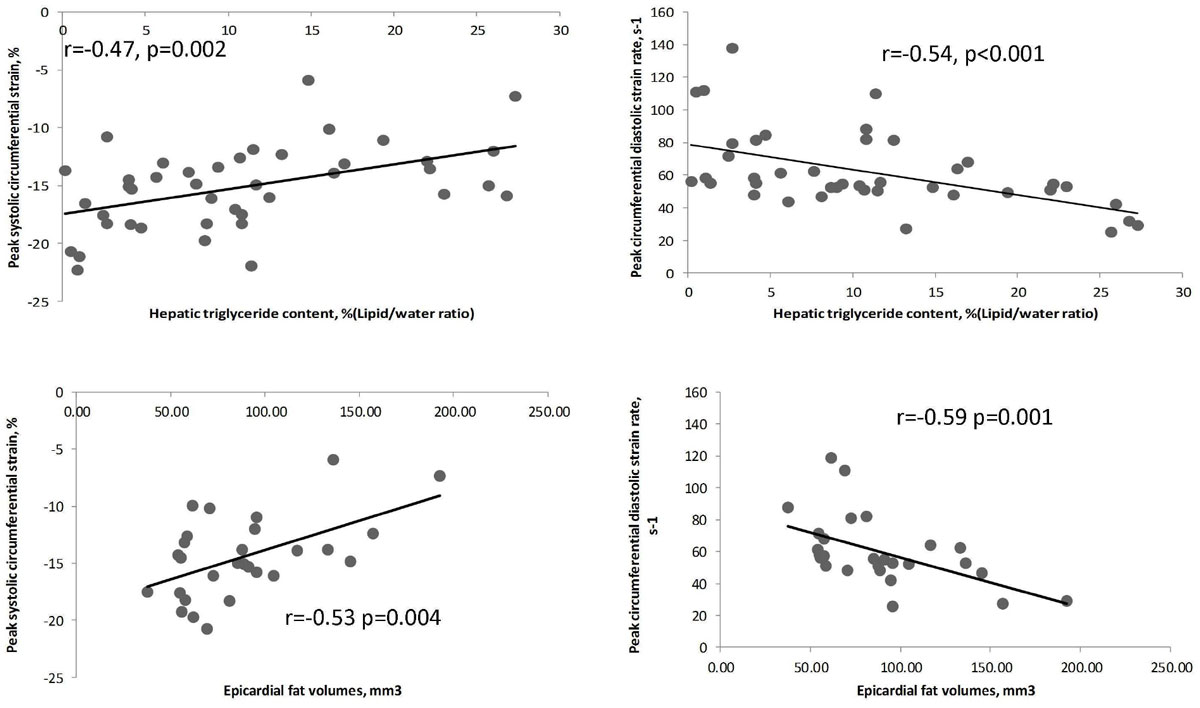
**Correlations of HTG and epicardial fat volumes with circumferential systolic and diastolic rates**.

Demographic, biochemical and multiparametric MRI results are provided in Figure 1.

When comparing L-T2DM to controls, diabetes, even in the absence of obesity, was associated with increased LV mass (p=0.03), impaired myocardial energetics (p=0.04), increased MTG (p=0.01) and HTG (p=0.04). While cardiac structural changes, and abnormalities in MTG and PCr/ATP were similar between the two T2DM groups, epicardial fat volumes (p=0.04) and HTG (p=0.01) were significantly increased in O-T2DM patients compared to L-T2DM. Moreover, HTG and epicardial fat volumes correlated negatively with the peak systolic circumferential strain and diastolic strain rates (Figure 2 for all), and in line with this, these functional changes were only impaired in O-T2DM patients (p < 0.001 and p = 0.006 respectively compared to controls), supporting a potential mechanistic role of ectopic adiposity for cardiac dysfunction in T2DM. MTG did not correlate with HTG or epicardial fat volumes. Finally, fibroinflammatory liver disease (elevated cT1) was also only evident in O-T2DM (p=0.004 and p < 0.001 vs L-T2DM patients and controls, respectively), and liver cT1 also correlated with HTG and epicardial fat volumes (p < 0.001 and p = 0.01 respectively).

**Table 1:** Demographic, biochemical and multiparametric MRI results.

	Controls N=12	Lean T2DM patients N=15	Obese T2DM patients N=27	P value
Age, y	50 ± 10	56 ± 9	56 ± 8	0.16
BMI, kg/m^2^	23 ± 3	23 ± 2	33 ± 3*	<0.001
Male,%	58	60	41	0.41
Diabetes Duration, years	...	6.1 ± 4.7	6.6 ± 6.5	0.78
Glycated hemoglobin, %	...	7.4 ± 0.9	7.7 ± 1.4	0.22
ALT, IU/L	22 ± 9	30 ± 22	36 ± 17	0.12
**Multiparametric Liver MRI**				
cT1,ms	753 ± 45	821 ± 67	924 ± 116*	<0.001
Hepatic triglyceride content, % (Lipid/water ratio)	3.6 ± 3.5	7.7 ± 4.6	14.8 ± 8.4*	<0.001
**CMR and Cardiac MRS Findings**				
LV end-diastolic volume, ml	145 ± 40	124 ± 33	126 ± 25	0.15
LV mass, g	98 ± 26	123 ± 33†	119 ± 28†	0.01
LV mass to LV end-diastolic volume, g/ml	0.63 ± 0.13	0.95 ± 0.26	0.89 ± 0.20	<0.001
Peak systolic circumferential strain, negative (-),%	18.1 ± 2.1	16.5 ± 2.6†	13.4 ± 3.6*	<0.001
Peak circumferential diastolic strain rate, s-1	74 ± 20	68 ± 19*	56 ± 26†	0.006
Mycocardial PCr/ATP ratio	2.08 ± 0.40	1.75 ± 0.29*	1.64 ± 0.32*	0.003
Mycocardial triglyceride, %(Lipid/water ratio)	0.48 ± 0.28	1.14 ± 0.66†	1.22 ± 0.91†	0.02

Values are mean ± standard deviations or percentages. T2DM indicates type 2 diabetes mellitus; CMR, cardiac magnetic resonancel cT1, corrected T1; ms, milliseconds; PCr, Phs phocreatine *p < 0.05 vs controls and lean T2Dm †p < 0.05 vs controls

## Conclusions

We demonstrate here, for the first time, not only that ectopic adiposity is more pronounced in obese compared to lean T2DM patients, but that it is also linked to cardiac contractile dysfunction and fibroinflammatory liver disease. However, myocardial steatosis is not associated with epicardial/hepatic adiposity, and may thus represent a separate entity influenced by factors beyond ectopic adiposity. Ectopic adiposity represents an important therapeutic target, and the reversal of body fat distribution abnormalities may improve cardiac function and prognosis in patients with diabetes.

